# A survey of access to trial of labor in California hospitals in 2012

**DOI:** 10.1186/1471-2393-13-83

**Published:** 2013-04-03

**Authors:** Mary K Barger, Jennifer Templeton Dunn, Sage Bearman, Megan DeLain, Elena Gates

**Affiliations:** 1 Department of Family Health Care Nursing, University of California, San Francisco, San Francisco, CA, USA; 2 University of California, Hastings San Francisco, CA, USA; 3 Department of Family Health Care Nursing, University of California, San Francisco, CA, USA; 4 UCSF/UC Hastings Consortium on Law, Science, & Health Policy, San Francisco, CA, USA; 5 Department of Obstetrics, Gynecology, and Reproductive Sciences, University of California, San Francisco, San Francisco, CA, USA

**Keywords:** Trial of labor after cesarean, Vaginal birth after cesarean, Access to care

## Abstract

**Background:**

In 2010, the NIH and ACOG recommended increasing women’s access to trial of labor after cesarean (TOLAC). This study explored access to TOLAC in California, change in access since 2007 and 2010, and characteristics of TOLAC and non-TOLAC hospitals.

**Methods:**

Between November 2011 and June 2012, charge nurses at all civilian California birth hospitals were surveyed about hospitals’ TOLAC availability and requirements for providers. VBAC rates were obtained from the California Office of Statewide Health Planning and Development (OSHPD). Distance between hospitals was calculated using OSHPD geocoding.

**Results:**

All 243 birth hospitals that were contacted participated. In 2010, among the 56% TOLAC hospitals, the median VBAC rate among TOLAC hospitals was 10.8% (range 0-37.3%). The most cited reason for low VBAC rates was physician unwillingness to perform them, especially due to the requirement to be continually present during labor. TOLAC hospitals were more likely to be larger hospitals in urban communities with obstetrical residency training. However, there were six (11.3%) residency programs in non-TOLAC hospitals and 5 (13.5%) rural hospitals offering TOLAC. The majority of TOLAC hospitals had 24/7 anesthesia coverage and required the obstetrician to be continually present if a TOLAC patient was admitted; 17 (12.2%) allowed personnel to be 15-30 minutes away. TOLAC eligibility criteria included one prior cesarean (32.4%), spontaneous labor (52.5%), continuous fetal monitoring and intravenous access (99.3%), and epidural analgesia (19.4%). The mean distance from a non-TOLAC to a TOLAC hospital was 37 mi. with 25% of non-TOLAC hospitals more than 51 mi. from the closest TOLAC hospital.

In 2012, 139 hospitals (57.2%) offered TOLAC, 16.6% fewer than in 2007. Since 2010, five hospitals started and four stopped offering TOLAC, a net gain of one hospital offering TOLAC with three more considering it. Only two hospitals cited change in ACOG guidelines as a reason for the change.

**Conclusions:**

Despite the 2010 NIH and ACOG recommendations encouraging greater access to TOLAC, 44% of California hospitals do not allow TOLAC. Of the 56% allowing TOLAC, 10.8% report fewer than 3% VBAC births. Thus, national recommendations encouraging greater access to TOLAC had a minor effect in California.

## Background

Since 1996 the number of women undergoing a trial of labor after cesarean (TOLAC) has dropped sharply, due in part to two sets of guidelines issued by the American College of Obstetricians and Gynecologists (ACOG). Following a widely publicized study [1] and increasing malpractice concerns around TOLACs [2-4], ACOG in 1998 revised its guidelines for women desiring a vaginal birth after a cesarean. The 1998 guideline differed from the earlier 1994 guideline by limiting TOLAC to women with one or two prior cesareans but kept in place the previous requirement that a physician capable of performing a cesarean should be “readily available” and that anesthesia be available [5]. Then, in 1999, ACOG re-issued its guideline in which the only changes were to replace physicians be ‘readily available’ with “immediately available”, and stipulated that 24-hour in-hospital anesthesia should also be available [6]. In the wake of these guidelines, the cesarean delivery rate in the United States rose from 21% to 32.8% between 1996 and 2010 [7] and the vaginal birth after cesarean (VBAC) rate (per 100 women with a prior cesarean) dropped from 28% to 8% [8]. In response to the rising cesarean rate, the decline in VBAC, and the intense focus on a rare outcome (0.5% for uterine rupture among TOLAC women) [9], the National Institutes of Health (NIH) convened a Consensus Development Conference Panel in March 2010 to address key questions surrounding the practice of TOLAC. A systematic literature review by a panel of experts showed that for women with one or two prior low transverse uterine incisions, both TOLAC and elective repeat cesarean delivery carry important risks and benefits, which differ for the woman and the fetus [10]. Given the available evidence, the Panel concluded that TOLAC “is a reasonable option for many pregnant women” and that efforts are needed to ensure that women with a prior cesarean are supported in making informed decisions about trial of labor versus an elective repeat cesarean [11]. “When [TOLAC] and elective repeat cesarean delivery are medically equivalent options”, the Panel’s statement encourages a shared decision-making process that, whenever possible, allows the woman’s preference to be honored [11].

Moreover, applying the “immediately available” standard only to VBAC patients is not warranted when other unforeseen obstetrical emergencies, such as placental abruption or prolapsed cord, occur at a higher incidence than uterine rupture in TOLAC [9,11,12]. Thus, the Panel recommended that ACOG revise its “immediately available” standard “with specific reference to other obstetrical complications of comparable risk, risk stratification, and in light of limited physician and nursing resources” [11].

In response, ACOG changed its clinical guidelines in August 2010 to reflect some of the NIH recommendations, stating that TOLAC was an appropriate choice for women with one or two previous low transverse cesareans, that labor induction should remain an option for women attempting TOLAC, and that twin gestation was not a contraindication for TOLAC [13]. However, ACOG preserved language recommending that TOLAC occur only in facilities with staff “immediately available” to perform an emergency cesarean, while acknowledging that this recommendation was based on Level-C evidence, expert opinion [13]. ACOG did recognize that this may not feasible in all facilities and should not be a reason to deny a woman the choice of TOLAC or force her to have a repeat cesarean. In situations where resources are less than optimal, or transfer to a facility that supports TOLAC is untenable, ACOG states that “respect for patient autonomy supports the concept that patients should be allowed to accept increased levels of risk, however, patients should be clearly informed of such potential increase in risk and management alternatives” [13].

Since the NIH Conference on VBAC published its findings encouraging hospitals to decrease barriers to TOLAC and ACOG issued its statement in August 2010 encouraging less restrictive TOLAC guidelines, we wanted to explore whether these two high profile publications have had any effect on the proportion of hospitals in California that limit or prohibit TOLAC. We also wanted to identify characteristics of hospitals that do and do not offer TOLAC and estimate the distance women in California would have to travel to undergo a TOLAC if their community hospital did not allow access to TOLAC.

## Methods

### Identifying TOLAC and non-TOLAC birth hospitals

This study employed a cross-sectional survey of California hospitals conducted between October 2011 and June 2012. We identified 243 civilian hospitals with labor and delivery units (“birth hospitals”) using 2010 birth statistics from a master list of hospitals available from the California Office of Statewide Health Planning and Development (OSHPD). This represents 97.8% of births among the 251 known civilian birth hospitals identified from a separate birth database [14]. To assess the degree to which the birth hospitals did or did not offer TOLAC, we compared the hospitals’ respective 2010 VBAC rates (the number of vaginal births per 100 women with a prior cesarean), found in the OSHPD Hospital Patient Data Utilization Rates for Selected Medical Hospital Procedures. Although it would have been preferable to use TOLAC rates, they are not readily accessible; research studies show that between 60-80% of women attempting TOLAC are successful, although this rate can be affected by individual factors [15-18]. Since the most recent data were from 2010, we omitted from the TOLAC category any hospitals that reported starting to offer TOLACs in 2010 or later. Likewise, we omitted from the non-TOLAC group any hospitals that stopped offering TOLAC in 2010 or later.

### Contacting the hospitals

A trained maternity nurse research assistant called all birth hospitals. Upon reaching the central hospital operator, she asked for the labor and delivery unit and, once connected, requested to speak to the nurse in charge. If the nurse in charge was not available, the research assistant would call back up to three times. If the nurse in charge was available, the research assistant described the study, including the estimated time needed to complete the survey (10-15 minutes) and obtained the nurse’s verbal consent to participate. If the nurse had time to speak, she was interviewed then. If she was too busy, the research assistant arranged a time to call back. For convenience, all nurses were given the option of taking the survey online, but none chose that method.

### Survey questions

Nurses were asked about the availability of TOLAC. If the hospital did not currently offer TOLAC, they were asked when the hospital last offered TOLAC, what were the hospital’s reasons for discontinuing TOLAC, and where they would direct a woman seeking TOLAC. In addition, the nurses were asked if the hospital had plans to offer TOLAC in the near future and if so, what factors prompted this policy change. If the hospital did offer TOLAC, the nurse was asked when it began doing so, if it was after 2006 (since this was the year with the latest data on approximately how many hospitals offered TOLAC), and what factors led to the policy change. We then asked whether the hospital had different labor policies for women having TOLAC than for other obstetrical patients, such as different requirements for physician and anesthesia availability.

We asked all 243 birth hospitals about the type of obstetrical providers on staff and the hospital’s availability requirements (in-hospital or on-call), as well as the presence of an emergency department and anesthesia services in the hospital. The research assistant also recorded verbatim or summary comments offered by the nurses regarding their practices or other obstetric personnel issues.

### Characterizing the hospitals

We then used the OSHPD database to further characterize the hospitals by ownership and geography. Annual number of births in each hospital although collected by OSHPD were only available from a separate state birth database. We classified a hospital as “public” if ownership was listed as a city, county, or district government; “private” if ownership was listed as an “investor” (corporate, individual, partnership, or limited liability company); and “nonprofit” if ownership was listed a non-profit (including religious and university hospitals) or the University of California. OSHPD classifies hospital setting using geographic units from the California Medical Service Study Area (MSSA): “urban” has at least 75,000 people and is no smaller than five square miles; “rural” has a population density of less than 250 persons per square mile; and “frontier” has a population density of less than 11 persons per square mile. Since only two birth hospitals were classified as “frontier”, we dichotomized the geographic variable to urban or rural, including “frontier” hospitals in the rural category. We used the designations from 2000 since new designations from the 2010 census were not yet available. OSHPD complete birth numbers by hospital were available for 2009. Using these numbers, we categorized hospitals with 1-1000 births as “small”, 1001-2500 births as “medium”, and more than 2500 births as “large”, allowing us to analyze three approximately equal-size groups.

We also characterized the hospitals by the sociodemographic makeup of their patients using the OSHPD 2010 Patient Discharge Data Inpatient file. We captured the race/ethnicity and payer information by looking at patients discharged with a childbirth code (MDC=14). To capture the percentage of English-speaking patients, we looked at *all* inpatient discharges since this variable was not available at a more precise level. For the three hospitals without 2010 discharge data, we used data from 2009.

### Distance to TOLAC

We used the hospitals’ location information, available from OSHPD, and ARCGIS 10.1 software (ESRI, Redlands, CA) to calculate and map the distances women would have to travel to obtain TOLAC if it was not offered by the hospital in their community. The distances refer to a straight line between two hospitals rather than actual travel distance on available roads. If a nurse identified a referral hospital that did not permit TOLAC or the identified hospital only accepted women with the hospital’s insurance, e.g., Kaiser Foundation hospital, then we substituted the nearest TOLAC hospital in our calculations.

### Statistical approach

Differences in hospital and provider characteristics between TOLAC and non-TOLAC hospitals were calculated using t-tests for continuous variables and chi-square for categorical ones. Analysis was done using SAS 9.2 PC software (SAS Institute, Cary, NC).

### Consent

University institutional review board approval was obtained prior to the start of the study. All participation was voluntary and we assured participants that we would maintain the anonymity of the survey respondent and the hospital.

## Results

### VBAC rates in California

California has about 500,000 births a year. Among the non-military hospitals, the 83,597 women with a prior cesarean had 6,855 vaginal deliveries for a rate of 8.2 per 100 deliveries of women with a prior cesarean [19]. Of the 243 birth hospitals we identified, all had a nurse in charge who completed our survey and 139, or 57.2%, stated they offered TOLAC. Among the hospitals that said they offered TOLAC in 2010, the 2010 VBAC rate ranged from zero to 37.3%, with a median of 10.8%. Seven TOLAC hospitals in the lowest fifth percentile had VBAC rates between 0 and 1%, and another seven in the next fifth percentile had rates between 1 to 2%. On the other hand, the VBAC rate for hospitals that did not offer TOLAC in 2010 ranged from 0 to 7.1%, with a median of 0.7%. Eighteen percent of non-TOLAC hospitals had VBAC rates greater than 2%.

### Hospital characteristics

The sociodemographics of the hospitals are found in Table 1. The birth hospitals offering TOLAC were larger and predominantly found in urban settings. The number of births at small hospitals offering TOLAC ranged from 200 to 990 with seven hospitals performing less than 600 births annually. Compared to non-TOLAC hospitals, the women they served were significantly less likely to have public insurance. The proportion of patients who spoke English was the same in TOLAC and non-TOLAC hospitals. Among the 37 designated rural hospitals with birth units, five (13.5%) offered TOLAC.

**Table 1:** Characteristics of California hospitals offering and not offering trial of labor after cesarean (TOLAC)

	**TOLAC hospital (N=139) N (%) or Mean (SD)**	**Non-TOLAC hospital (N=104) N (%) or Mean (SD)**	**p-value**
Hospital Size			<0 .0001
Small (0-1000 births)	17 (12.2)	53 (50.9)	
Medium (1001-2500 births)	53 (38.1)	37 (35.6)	
Large (>2500 births)	69 (49.6)	14 (13.5)	
Ownership Type			0.060
Public	20 (14.4)	23 (22.1)	
Non-profit	98 (70.5)	58 (55.8)	
For profit	21 (15.1)	23 (22.1)	
Geographic location			<.0001
Urban	134 (96.4)	72 (69.2)	
Rural	5 (3.6)	32 (30.8)	
Percent with public insurance	46.66 (27.8)	60.63 (23.0)	<.0001
Percent English speaking	83.57 (13.6)	85.98 (13.0)	0.163

### Availability of providers & anesthesia in the hospitals

Several variables related to provider type and availability requirements differed between TOLAC and non-TOLAC hospitals. While 242 of the 243 birth hospitals had obstetricians on staff, only 53 (21.8%) had obstetric resident training which in 5 cases occurred in non-TOLAC hospitals (see Table 2). In fact, one nurse cited the establishment of an obstetric residency program as leverage the hospital used to negotiate with its insurer to allow TOLAC. Nurse-midwives were more likely to have delivery privileges at hospitals offering TOLAC. Compared to non-TOLAC hospitals, TOLAC hospitals were six times more likely to have a system to ensure an obstetric provider be in the hospital all of the time if they had any woman in labor, regardless of cesarean history (46.3% versus 6.9% in non-TOLAC hospitals). Of the 64% of TOLAC hospitals that did not require providers to be in-hospital, the vast majority (91.9%) required a provider to be within 15 minutes of the hospital. At least three nurses noted that the presence of an obstetric hospitalist facilitated their ability to allow TOLAC. Anesthesia coverage was available 24 hours a day/7 days a week in 110 (79.1%) of TOLAC hospitals, compared to 30 (29%) of non-TOLAC hospitals. For the TOLAC hospitals in which anesthesia was present during the day but on-call, not in-hospital, at least part of the time, such as on the weekends, more than 90% were required to be available within 15 minutes. Virtually all birthing hospitals had an emergency department.

**Table 2:** Hospital provider types and policies by TOLAC hospital status

	**TOLAC hospital (N=139) N (%) or Mean (SD)**	**Non-TOLAC hospital (N=104) N (%) or Mean (SD)**	**p-value**
**Type of provider**			
Obstetrician	139 (100.0)	103 (99.0)	0.2476
Obstetric Resident Program	47 (33.8)	6 (5.8)	<0.0001
Family Practice Physician	45 (32.4)	25 (24.0)	0.1566
Nurse-midwife	53 (38.1)	22 (21.1)	0.0047
**OB Provider in-hospital if any laboring patient**	63 (46.3)	7 (6.9)	<0.0001
*Missing*	*3*	*2*	
**If not in-house, how far away**			
Within 15 minutes	67 (91.8)	64 (68.1)	
Within 30 minutes	6 (8.2)	30 (31.9)	
*Missing*	*3*	*3*	
**Anesthesia coverage**			<0.0001
24/7 in-hospital	110 (79.1)	30 (29.1)	
Weekday in-hospital/ night or weekend on-call	26 (18.7)	59 (57.3)	
On-call	3 (2.2)	14 (13.6)	
If on call, time to respond			
Within 15 minutes	26 (92.9)	56 (75.7)	
Within 30 minutes	2 (7.1)	15 (20.3)	
Greater than 30 minutes	0 (0.0)	3 (4.0)	
**Hospital has emergency room**	138 (99.3)	101 (98.1)	0.3965
*Missing*	*1*	*3*	

In nearly one-third of the non-TOLAC hospitals, anesthesia was available 24 hours a day. Of the seven non-TOLAC hospitals that require the obstetric provider to be in the hospital throughout labor, three have 24 hour anesthesia coverage and the other four have anesthesia available during the day, but on-call nights and/or weekends. In addition, of the 64 non-TOLAC hospitals where the provider must be within 15 minutes of the hospital, 21 have 24/7 anesthesia coverage. Therefore, 24 to 28 non-TOLAC hospitals have the potential to offer TOLAC and still satisfy ACOG’s “immediately available” criteria. In one case, a hospital that recently stopped allowing TOLAC had a 2010 VBAC rate above 10% and had all the elements needed to comply with ACOG guidelines but did not want to give up operating time for planned cesareans if a woman with a TOLAC was admitted. According to the nurses surveyed, we found that about half of hospitals with continuous anesthesia coverage did not offer TOLAC, not because of an explicit hospital policy against it, but because physicians were unwilling to stay in the hospital with a woman attempting TOLAC.

Once a woman was admitted for TOLAC, the number of hospitals requiring physicians to be available in the hospital doubled to 87.3%, with the remainder requiring physicians to be available within 15-30 minutes (see Table 3). The number of hospitals requiring anesthesia presence was even higher at 93.5%. Virtually all hospitals had neonatal staff continuously available in the hospital. Only two hospitals had restrictions on the day of the week that a woman could undergo a TOLAC.

**Table 3:** Labor management policies when a woman was present attempting a TOLAC

	**TOLAC hospital (N=139) N (%) or Mean (SD)**
Anesthesia	
In-hospital	129 (93.5)
Within 30 minutes	9 (6.5)
*Missing=1*	
Physician	
In-hospital	117 (87.3)
Within 20-30 minutes	17 (12.7)
*Missing =5*	
Operating room immediately available	134 (96.4)
Neonatal staff available	136 (97.8)
Day of the week restrictions	
No restrictions	135 (97.1)
Day of the week	2 (1.4)
Time of day	0
Patient Restrictions	
Number of prior cesareans	131 (94.2)
One prior cesarean	45 (32.4)
No more than two prior cesareans	86 (61.8)
Singleton pregnancy	83 (59.7)
Time since last birth	11 (7.9)
Weight or body mass index	2 (1.4)
Spontaneous labor	73 (52.5)
Induction or labor management methods permitted	
Prostaglandin gel/insert	2 (1.4)
Foley	66 (48.9)
Oxytocin	128 (92.1)
Amniotomy	131 (94.2)
Requirements of women with a TOLAC	
Continuous fetal monitoring	138 (99.2)
Continuous IV	138 (99.2)
Epidural in active labor	27 (19.4)

### Patient TOLAC eligibility criteria

In a given hospital, restrictions on which women could undergo TOLAC included a combination of hospital policy and the standards of care adopted by the practicing physicians. Many nurses commented that a patient’s TOLAC eligibility was determined by physicians, rather than a stated hospital policy. The number of prior cesareans was a major eligibility criterion, with eligibility limited to one prior cesarean in 45 (32.4%) of hospitals and to two prior cesareans in 86 (61.8%) (Table 3). At least 60% of nurses responded that only a singleton pregnancy was appropriate for TOLAC. Two nurses agreed that there was a maximum weight or body mass index requirement. Eleven (7.7%) agreed there was a requirement for minimum time since the last cesarean.

In seventy-three (52.5%) of the hospitals, women were required to enter labor spontaneously in order to attempt TOLAC (Table 3). For the 66 (47.5%) hospitals in which induction of labor was allowed for women with TOLAC, all permitted amniotomy or foley catheters, 55 (83.3%) permitted use of oxytocin, and 2 (3.0%) allowed use of prostaglandin gel or inserts. However, among hospitals where induction of labor was not permitted for women attempting TOLAC, 85% permitted amniotomy and use of oxytocin for labor augmentation. There was a nearly universal requirement for both continuous fetal monitoring and continuous intravenous infusion during TOLAC, with only one hospital in which women did not have to have either. An epidural in active labor was a requirement in 27 (19.4%) hospitals.

### Distance for TOLAC access

The mean direct distance from a non-TOLAC hospital to a TOLAC hospital is 37 mi (59.4 km). The median distance is 20 mi (32.3 km), however, in this case, the actual travel distance by road is 6 mi (10 km) greater.

**Figure 1:**
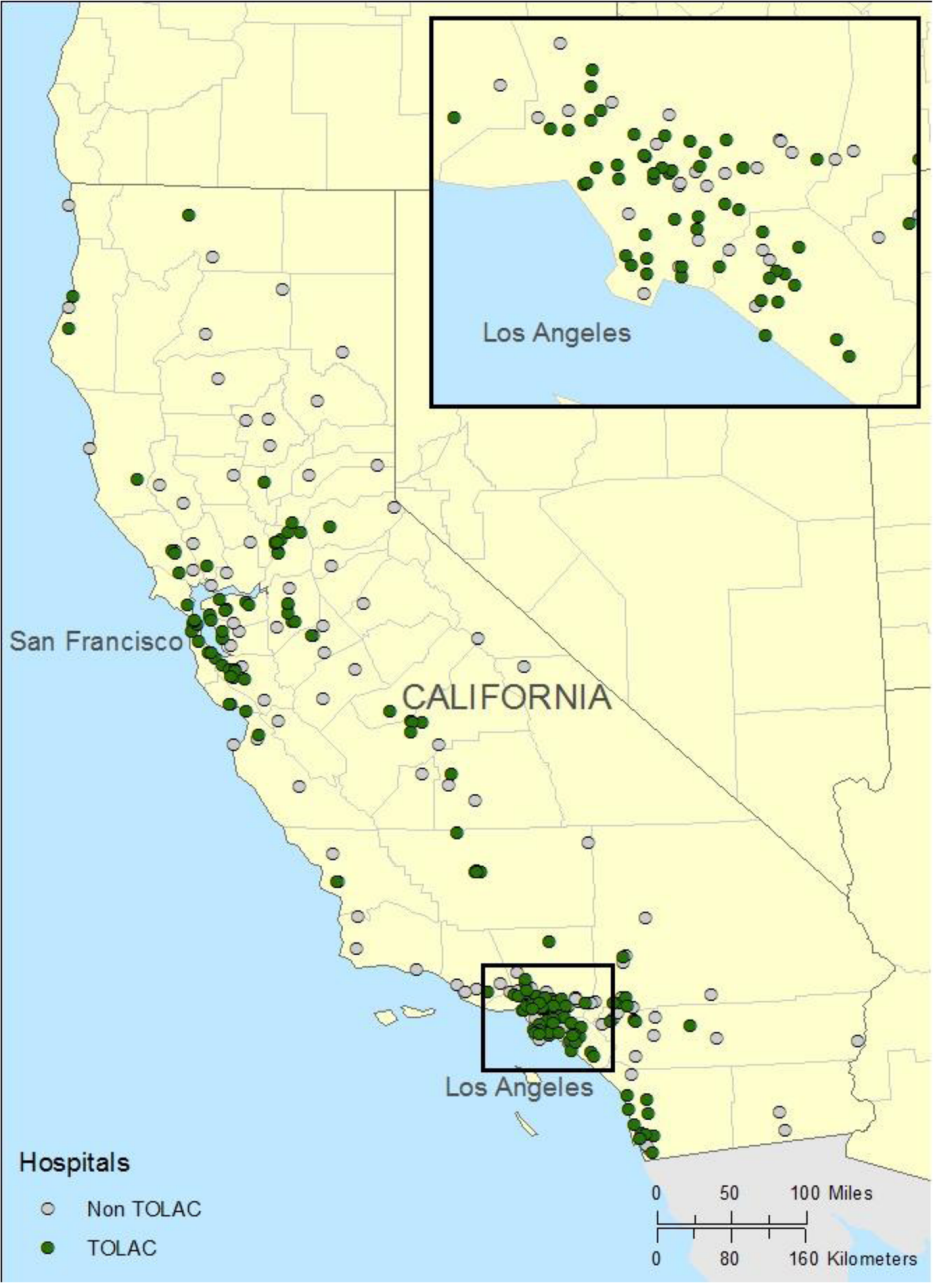
Geographical distribution of California birth hospitals that do and do not offer trial of labor after cesarean (TOLAC) in 2012.

The range of direct distance to the nearest TOLAC hospital was 0.5 to 195 mi (0.8 to 314.5 km), with 25% of non-TOLAC hospitals referring women to hospitals more than 52 mi. (83.5 km) away. Ten non-TOLAC hospitals were more than 91 mi. (152 km) from the closest TOLAC hospital (see Figure 1). In one case, although the nearest hospital was 186 mi. (310 km) away, the driving distance by road was actually 262 mi (421 km.) with a travel time of more than 5 hours.

#### Factors influencing hospitals to offer and not offer TOLAC

In our survey, 70 (67.3%) nurses from non-TOLAC hospitals could identify when the hospital stopped offering TOLAC. Forty hospitals stopped in or prior to 2004 and 24 of the remaining 30 stopped between 2005 and 2007. Between 2007 and 2011/2012, 12 hospitals (5% of all birth hospitals) stopped offering TOLAC: six in 2007; one per year 2008, 2009, and 2010; and three in 2011. Nurses were asked to identify one or more reasons why the hospital discontinued TOLAC. The most frequently cited reasons were anesthesia considerations and lack of immediate obstetrician availability (see Table 4). ACOG guidelines and hospital or corporate policy change were cited fairly equally (see Table 4). Three hospitals stated there were plans to start allowing TOLAC, with physician preference indicated as the reason for the change.

**Table 4:** Reasons nurses cited for hospitals stopping or starting to offer TOLACs*

**Reasons for stopping to offer TOLAC**	**Stopping (N=99) N(%)**	**Starting after 2007 (N= 10) N(%)**	**Reasons for starting to offer TOLAC**
Anesthesia not immediately available	70 (70.7)	3 (30.0)	Change in staffing: anesthesia available (2); adding hospitalist
Obstetrician not immediately available	54 (54.5)		
ACOG guidelines	48 (48.5)	2 (20.0)	ACOG Guideline change
Hospital or corporate policy change	44 (44.4)	1 (10.0)	Hospital or corporate policy change
Physician preference	26 (26.2)	10 (100.0)	Physician request
Other	17 (17.2)	1 (10.0)	Other: initiated OB residency
Liability or insurance	11 (11.1)	1 (10.0)	Insurance/liability changes
Operating room availability	18 (18.2)	7 (70.0)	Consumer demand

*Nurses could choose more than one reason. Denominator is hospitals where the nurses gave at least one reason.

Of the 139 hospitals offering TOLAC, 10 began doing so after 2007, with five starting in 2010 or later. Nurses were presented with eight possible reasons why the hospital changed its TOLAC policy. The most commonly cited reason was physician’s preference/physicians pushing for the change. Three nurses cited change in staffing, such as adding a hospitalist or availability of 24 hour anesthesia. Consumer demand was cited by seven nurses. None chose the 2010 NIH Vaginal Birth After Cesarean Conference Report or research studies but two chose the 2010 revision of the ACOG guidelines (see Table 4). In summary, four hospitals stopped and five started offering TOLAC from 2010 to present, for a net gain of one hospital offering TOLAC and three more making plans to do so. However, another perspective is that among the 166 California hospitals offering TOLAC in 2007, 16.6% fewer were offering this option to women five years later.

## Discussion

It is clear that in the last 9 years, access to TOLAC has become more restricted in California. A 2003-2004 hospital-based survey conducted of 225 (out of 268) California birth hospitals identified 26% that did not allow TOLAC [20]. By the time of our survey in 2011-2012, that percentage had jumped to 42.8% (N=104). The true picture of access to TOLAC may be even worse for two reasons. First, Kaiser Foundation, an integrated health management provider with its own hospitals accounted for one fourth of all VBAC births despite doing only 13% of the total births in California [21]. Secondly, the existence of a policy to *permit* TOLAC does not necessarily mean the hospital actually *provides* TOLAC. Among hospitals permitting TOLAC, the VBAC rates for 14 (10%) were below 2%. The difference between a hospital policy allowing TOLAC and actual TOLAC rate seems to be largely dependent on the willingness of obstetricians to perform VBACs. This might be best summarized by one nurse’s statement, “If a woman seeking a VBAC called here and asked if we do them, sure! But try calling around and finding a doc who actually will do the VBAC. That's trickier.” Among the 73 (52.5%) TOLAC hospitals where nurses offered spontaneous comments, over one-third said that less than a majority of the obstetricians attending at their hospitals would permit TOLAC for their patients, with 10 commenting that only one or two physicians would allow it. Physician reluctance to offer TOLAC is likely a combination of factors identified in the literature: fear of liability, previous experience with a uterine rupture from TOLAC, involvement with a cesarean related malpractice case, insurance carriers not allowing TOLAC, and convenience of scheduled repeat cesareans [22,23]. Two comments from the nurses surveyed summarize many of the issues. As one explained, “I've been here 22 years, and we used to do them a lot. But then we had so many abruptions, and just awful outcomes so the docs stopped wanting to do them. I think our hospital policy still says it's okay because we have in house anesthesia for our floor 24/7 but the doctors send all of the VBACs to [another hospital].” Another stated, “You know it's funny. We have 24/7 anesthesia, but only one medical group has the malpractice insurance to deliver VBACs here. And the docs don't want to stay on campus through the whole labor, so we aren't able to provide VBAC. Everyone always says we don't do it because of ACOG, but I'm realizing now that's not really true.” Another possible factor is physicians’ lack of exposure to TOLAC management during obstetrical residency, especially for physicians trained in the last 10 years, as VBAC rates have plummeted [22]. In our survey, six hospitals with obstetrical residents did not offer TOLAC. We do not know if these are the only hospitals in which these residents train but the lack of exposure to TOLAC management may be an important factor in the trainees’ willingness to allow women a TOLAC post-residency.

Did the 2010 policy statements from the NIH and ACOG make a difference? It is clear that ACOG’s more restrictive 1999 VBAC guidelines played a significant role in California, and probably nationally, in decreasing VBAC rates [24,25]. However, it seems that the most recent re-issue of ACOG guidelines has had, at most, a minor effect. From 2010 to present, five hospitals began offering TOLAC but four hospitals stopped offering this option, resulting in a net gain of one hospital offering TOLAC (with three more hospitals making plans to begin offering TOLAC). Moreover, none of the nurses in hospitals initiating TOLAC in 2010 or later identified the NIH VBAC report and only two cited the ACOG guideline change as a reason. Changes in staffing that allowed anesthesia and/or obstetrician immediate availability seemed to play a larger role, especially if one includes establishing an obstetric residency in this category. In at least three cases, nurses cited an obstetrician championing the change as one of the most important factors, speaking to the impact obstetricians can have in influencing practice. However, the change in ACOG guidelines and recommendations of the NIH may have bolstered their confidence to pursue a change. The other factor cited, but not spontaneously commented on, was consumer demand. We did not ask TOLAC hospitals when their current restrictions on which women were eligible for TOLAC started or were revised. However, the newest ACOG guidelines may have had limited effect since one-third still limit women to one previous cesarean and half require women go into spontaneous labor, two requirements contrary to the guidelines.

The issue of the “immediate availability” of a physician, which the newest ACOG statement chose to retain, despite being based on Level C evidence (expert opinion), is perceived as one reason for the decline in VBAC in the United States [26]. Limiting this standard to women attempting TOLAC has been questioned given the rarity of uterine rupture compared to other more common obstetrical emergencies. It is clear from our data, and others have noted [23], that “immediate availability” has a varied meaning among hospitals. Although a minority (13%) of hospitals consider a physician to be “immediately available” if capable of performing a cesarean within 20-30 minutes, most hospitals seem to have more stringent requirements for anesthesia and operating room availability. It was clear from a statement from one hospital that recently stopped offering TOLAC that having an empty operating room while a woman attempting a TOLAC was in labor meant giving up scheduled cesareans, something the obstetricians were not willing to do. What may be most informative is not that we found that larger, urban hospitals are more likely to offer TOLAC, but that there are small hospitals in rural communities that also offer them. Although they do not have the delivery volume to maintain anesthesia 24/7, they are able to put in place the resource team as outlined by Minkoff [12] to allow the option of TOLAC.

The need to support patient choice is clearly stated in the ACOG guidelines: “respect for patient autonomy supports the concept that patients should be allowed to accept increased levels of risk, however, patients should be clearly informed of such potential increase in risk and management alternatives” [13]. The comment of one nurse working in a rural setting captures this well, “We certainly do not advertise the fact that we do VBACs, but we have a cesarean refusal form that women sign if they are attempting TOLAC. …I know ACOG relaxed the rules around VBAC, but the wording can be so easily skewed. That's probably why we don't have an official policy for VBACs but instead have a cesarean refusal process.” Data from this study indicate that in some hospitals that do have the capacity to support TOLAC, very low VBAC rates and nursing comments reveal that it is effectively not allowed.

This study has some important limitations. We interviewed the nurse in charge on the day we called. It is possible that we may have gotten different responses if we had called on a different day and a different nurse responded. Certainly the qualitative comments would have been different. However, the process of asking the nurse in charge about whether TOLAC was allowed and if not, the reason/s it was not offered was the same one used by Shihady et al in their 2003-2004 California hospital TOLAC survey [20]. The nurse may not have been knowledgeable about changes in TOLAC policy that may have preceded their employment although we limited this inquiry to the last 6 years. No nurses spontaneously noted they had started working at the hospital after 2006. They also may not have been aware of all the reasons for a change in policy. Therefore, the 2010 statements may have played an important role in an administrative change but when the policy change was communicated to the level of charge nurses, it was either absent or not remembered by them. It is unclear if the responses would have been different if physicians or administrators were answering. They may have had alternative explanations for physician willingness to perform TOLACs. Unfortunately, some comments, such as the trade-off between TOLAC and having operating time for cesareans, came up spontaneously; it would be important in future similar research to obtain more systematic information about such potential tradeoffs.

## Conclusion

We conclude that where an individual provider or group of providers wants to advocate for a hospital to offer the option of TOLAC, national policy guidelines probably play an important supporting role, and change in practice can be accomplished. However, more research needs to be done to understand provider unwillingness to give women a choice in their delivery method in settings where there are no obvious institutional barriers. In the face of evidence supporting the safety of TOLAC and the positions reflected in NIH and ACOG publications, the apparent physician reluctance to support patient choice in method of delivery is difficult to justify.

## Competing interests

Authors have no financial or non-financial competing interests to disclose.

## Authors’ contributions

MKB conceived the study, coordinated data collection, performed all statistical analyses and drafted the manuscript. JTD participated in study design and assisted in data interpretation. SB conducted the study surveys and cleaned the study database. MD assisted in drafting the manuscript. EG participated in study design, data interpretation, and helped to draft the manuscript. All authors read and approved the final manuscript.

## Pre-publication history

The pre-publication history for this paper can be accessed here:

http://www.biomedcentral.com/1471-2393/13/83/prepub

## References

[B1] McMahon MJ Luther ER Bowes WA Jr Olshan AF Comparison of a trial of labor with an elective second cesarean section N Eng Med 1996 335 10 689 695 10.1056/NEJM199609053351001 8703167

[B2] Stalnaker BL Maher JE Kleinman GE Macksey JM Fishman LA Bernard JM Characteristics of successful claims for payment by the Florida Neurologic Injury Compensation Association Fund Am J Obstet Gynecol 1997 177 2 268 271 discussion 271-263 10.1016/S0002-9378(97)70186-8 9290439

[B3] Phelan JP VBAC: time to reconsider? OBG Manage 1996 8 62 68

[B4] Flamm BL Once a cesarean, always a controversy Obstet Gynecol 1997 90 2 312 315 10.1016/S0029-7844(97)00263-9 9241315

[B5] ACOG Practice Bulletin Vaginal birth after previous cesarean delivery 1998 Washington, DC: American College of Obstetricians and Gynecologists

[B6] ACOG Practice Bulletin Vaginal birth after previous cesarean delivery 1999 Washington, DC: American College of Obstetricians and Gynecologists

[B7] Martin JA Hamilton BE Ventura SJ Michelle M Osterman MJ Wilson EC Mathews TJ Births: final data for 2010 Nat Vit Stat Report 2012 61 1 1 100 24974589

[B8] Cox KJ Providers' perspectives on the vaginal birth after cesarean guidelines in Florida, United States: a qualitative study BMC Pregnancy Childbirth 2011 11 72 10.1186/1471-2393-11-72 21992871 PMC3203084

[B9] Guise JM Denman MA Emeis C Marshall N Walker M Fu R Janik R Nygren P Eden KB McDonagh M Vaginal birth after cesarean: new insights on maternal and neonatal outcomes Obstet Gynecol 2010 115 6 1267 1278 10.1097/AOG.0b013e3181df925f 20502300

[B10] Guise JM Eden K Emeis C Denman MA Marshall N Fu RR Janik R Nygren P Walker M McDonagh M Vaginal birth after cesarean: new insights Evid Rep Technol Assess (Full Rep) 2010 191 1 397 20629481 PMC4781304

[B11] Bangdiwala SI Brown SS Cunningham FG Dean TM Frederiksen M Hogue CJ King TL Lukacz ES McCullough LB Nicholson W NIH Consensus Development Conference Draft Statement on Vaginal Birth After Cesarean: New Insights NIH Consens State Sci Statements 2010 27 3 1 42 20228855

[B12] Minkoff H Fridman D The immediately available physician standard Semin Perinatol 2010 34 5 325 330 10.1053/j.semperi.2010.05.005 20869548

[B13] ACOG Practice Committee Vaginal birth after previous cesarean delivery (Number 115) Obstet Gynecol 2010 116 2 Pt 1 450 463 20664418 10.1097/AOG.0b013e3181eeb251

[B14] Community Perinatal Network Quality Improvement Data for California Perinatal Facilities and Regionshttp://www.perinatalnetwork.org/

[B15] Coassolo KM Stamilio DM Pare E Peipert JF Stevens E Nelson DB Macones GA Safety and efficacy of vaginal birth after cesarean attempts at or beyond 40 weeks of gestation Obstet Gynecol 2005 106 4 700 706 10.1097/01.AOG.0000179389.82986.50 16199624

[B16] Grobman WA Rates and prediction of successful vaginal birth after cesarean Semin Perinatol 2010 34 4 244 248 10.1053/j.semperi.2010.03.003 20654774

[B17] Landon MB Hauth JC Leveno KJ Spong CY Leindecker S Varner MW Moawad AH Caritis SN Harper M Wapner RJ Maternal and perinatal outcomes associated with a trial of labor after prior cesarean delivery N Eng Med 2004 351 25 2581 2589 10.1056/NEJMoa040405 15598960

[B18] Macones GA Peipert J Nelson DB Odibo A Stevens EJ Stamilio DM Pare E Elovitz M Sciscione A Sammel MD Maternal complications with vaginal birth after cesarean delivery: a multicenter study Am J Obstet Gynecol 2005 193 5 1656 1662 10.1016/j.ajog.2005.04.002 16260206

[B19] State of California Office of Statewide Health Planning and Development Utilization rates for selected medical procedures in California 2010 http://www.oshpd.ca.gov/HID/Products/PatDischargeData/ResearchReports/HospIPQualInd/Vol-Util_IndicatorsRpt/index.html

[B20] Shihady IR Broussard P Bolton LB Fink A Fridman M Fridman R Aydin C Korst LM Gregory KD Vaginal birth after cesarean: do California hospital policies follow national guidelines? J Reprod Med 2007 52 5 349 358 17583231

[B21] Center for Health Statistics California Births 2010 California Department of Public Health http://www.cdph.ca.gov/data/statistics/Pages/StatewideBirthStatisticalDataTables.aspx

[B22] Wells CE Vaginal birth after cesarean delivery: views from the private practitioner Semin Perinatol 2010 34 5 345 350 10.1053/j.semperi.2010.05.008 20869551

[B23] Charles S The ethics of vaginal birth after cesarean Hastings Cent Rep 2012 42 4 24 27 22777976 10.1002/hast.52

[B24] Menacker F Declercq E Macdorman MF Cesarean delivery: background, trends, and epidemiology Semin Perinatol 2006 30 5 235 241 10.1053/j.semperi.2006.07.002 17011392

[B25] Zweifler J Garza A Hughes S Stanich MA Hierholzer A Lau M Vaginal birth after cesarean in California: before and after a change in guidelines Ann Fam Med 2006 4 3 228 234 10.1370/afm.544 16735524 PMC1479438

[B26] Menacker F Hamilton BE Recent trends in cesarean delivery in the United States NCHS Data Brief 2010 35 1 8 20334736

